# Avatars and humans may not elicit the same accent-related biases in mock courtroom research

**DOI:** 10.3389/fpsyg.2025.1459044

**Published:** 2025-02-07

**Authors:** Lara A. Frumkin, Anna Stone, Mary Jane Spiller

**Affiliations:** ^1^School of Psychology & Counselling, The Open University, Milton Keynes, United Kingdom; ^2^Department of Psychology and Human Development, School of Childhood and Social Care, University of East London, London, United Kingdom

**Keywords:** avatars, accent, eyewitness, country of origin, courtrooms

## Abstract

**Introduction:**

Conducting research to better understand the role of extralegal factors in courtroom decision-making requires either labor intensive methods, such as simulating a trial, or approaches that are not ecologically valid, such as using short written case vignettes. If avatars could be used in simulated courtrooms, experiments could more easily manipulate extralegal variables for study without requiring significant resourcing, for example hiring actors and having access to a courtroom. The current study used previously developed stimulus materials of a human eyewitness in a courtroom and created a comparable avatar eyewitness and virtual courtroom to assess ratings of the human and avatar.

**Method:**

Participants (*N* = 703) saw one of 12 videos depicting an eyewitness on the stand at a criminal trial recounting a burglary. The design was a 2 × 2 × 3, mode of presentation (human or avatar), accent (General American English or non-standard) and country of origin (Germany, Mexico or Lebanon). Three actors voiced each human and avatar pair using General American English and one of the non-standard accents (German, Mexican or Lebanese) so that variation in ratings could be attributed to presentation mode, accent and country of origin.

**Results:**

An analysis of covariance revealed that the avatar witnesses were rated more favorably than the humans and there were no main effects of accent nor country of origin, contrary to previous research using the human video stimuli. A three-way interaction showed the Lebanese human non-standard accented witness was rated more poorly than her standard-accented counterpart, her avatar counterpart, and the Mexican and German human non-standard accented witnesses.

**Discussion:**

Findings reveal that avatar witnesses cannot yet reliably replace their human counterparts. Discussion as to what can be done in future to further investigate how to create courtroom stimulus materials is presented along with possible explanations as to the reasons for different findings in this research than previous studies.

## 1 Introduction

Fairness and equality are at the heart of the justice system. It is, therefore, important to understand the nature and impact of implicit prejudices that may arise when ordinary citizens with no legal training hear evidence and make decisions. Juries conduct their deliberations in secret, so research into jury decision-making has to use mock juries and trials. Developing credible courtroom scenarios is resource consuming, usually requiring access to a courtroom and actors. The current study aimed to explore the use of avatars as proxies for human actors in mock trial scenarios. An avatar, for the purpose of this research, is defined as a computer-generated representation of a human. If avatars could be used, a range of potentially relevant extralegal factors could readily be studied, providing experimental evidence to the criminal justice system on what implicit biases might contribute to legal decision-making processes (e.g., Bornstein and Kleynhans, 2018). Before recommending the use of avatars in jury decision-making research, we need to understand whether an avatar will provoke the same range and nature of responses as would be expected from a human. Accent was chosen as the variable used to elicit a range of responses, based on prior research showing that participants in mock-courtroom settings respond differently to varying accents (e.g., Cantone et al., 2019; Paver et al., 2025). The use of a well-understood factor enables the research to focus on the differences in the perceptions of avatars and humans.

Importantly, several studies have reported results suggesting that the perception of avatars has similar effects to the perception of humans, suggesting their use in court room studies may be viable. For example, Leding et al. (2015) reported that after viewing un/attractive avatars, participants evaluated real human faces as more/less attractive. This observation of the standard contrast effect supports the proposition that human-like avatars will provoke similar responses to real humans in a situation of passive viewing, as would be the case with mock courtroom studies. de Borst and de Gelder (2015) argued that in studies using computer-generated stimuli, emotional expressions were perceived similarly on the faces of human-looking avatars and humans. The proposition that avatars may be perceived similarly to humans is consistent with the view taken by Donath (2007, p. 53) that when an interface uses human-like avatars, “the issue of user interpretation of character traits […] is inevitable.”

Furthermore, studies exploring online interactions with avatars have reported results suggesting the perception of human characteristics in human-like avatars. These include similar perceptions of credibility (Nowak and Rauh, 2008); interpersonal distance and eye contact (Yee et al., 2007); sense of social presence of others (Felnhofer et al., 2018); and reactions to being ostracized (Kothgassner et al., 2017) when viewing human-like avatars and humans.

There are, however, reservations concerning the extent of character trait similarities inferred from humans and those from avatars. Interpretating character traits from human-like avatars (Donath, 2007) does not necessarily imply viewers would have identified the same character traits in humans, with some research showing differential information processing of the human and avatar (Riedl et al., 2011). MacDorman and Ishiguro (2006, p. 297) offer a cautionary view that “preliminary results indicate that only very humanlike devices can elicit the broad range of responses that people typically direct toward each other.”

The evidence that people frequently respond to online avatars as they might respond to human agents offers the opportunity for the use of lifelike avatars to provide a resource-light avenue for exploring the influence of extralegal factors in psychology courtroom research. However, without direct comparisons, it is unclear if humans and avatars in mock courtroom trials will be perceived equally.

What remains to be answered are two questions: first, whether we rate humans and avatars, with the same pose, movements, ethnicity, and degree of attractiveness, equally when they present the same evidence to viewers; and second, whether similar patterns of bias, based on accent in the current research, would be observed for human and avatar witnesses. This is particularly important in the high stakes environment of the courtroom. Turning to research on accents and country of origin (CO), for which there are predicted effects, we will investigate whether human and avatar witnesses elicit the same perceptions from participants.

Non-standard accents are typically spoken by members of minority groups, those from lower socioeconomic status backgrounds and non-native speakers (Fuertes et al., 2012). A standard, or mainstream accent, such as news anchor English [General American English (GAE) or General British] is typically perceived more favorably than non-standard and foreign accents (Carlson and McHenry, 2006; Dragojevic and Giles, 2016; Giles and Coupland, 1991; Seggie, 1983) perhaps due to negative attitudes people hold of non-standard accented speakers (Bresnahan et al., 2002; Dragojevic et al., 2017). Coupland and Bishop’s (2007) UK study supports this, finding participants rated a GAE accent 8th on prestige out of 34 accents and German rated 23rd.

Turning to directly relevant courtroom research, UK mock defendant research has shown that standard speakers are seen as less guilty than those with non-standard dialects (Dixon et al., 2002). Kurinec and Weaver’s (2019) US research provides evidence that non-standard speakers, with African American Vernacular English (AAVE), were rated less well and received a greater number of guilty verdicts when compared with standard GAE speakers. Similar findings were offered by Cantone et al. (2019) supporting the idea that defendants with standard accents are thought of more favorably. Research has also shown eyewitness accent can impact favorability impressions of speakers. Frumkin and Thompson (2020) compared General British speech, Multicultural London English (MLE; local London regional accent) and a Birmingham accent using a matched-guise technique whereby one person produces speech in two or more accents and found the standard speakers were considered more accurate, credible and prestigious than either of the non-standard regional accents. Another study evidenced that General British speakers were more favorably rated and viewed as having higher levels of education and occupational status than non-standard speakers (Frumkin and Stone, 2020).

These findings support stereotype content models (SCM) (see for example Cuddy et al., 2009) such as accent prestige theory (APT) (Anderson et al., 2007; Fuertes et al., 2002) which theorizes that accent is an important feature in determining respect based on speech. APT is made up of two dimensions: status, including intelligence, education, and social class, and solidarity, consisting of friendliness, trustworthiness, and kindness. Status speakers typically have standard, high-status accents and are likely be rated well. Solidarity speakers are rated well by listeners with similar accents to the speaker. This points to the concept of assimilation such that a witness with a standard GAE accent would be perceived to be well assimilated, with an accent familiar to listeners, and therefore given higher ratings (Uzun, 2023). The present research was designed to investigate whether these accent-related perceptions would be elicited equally by avatar and human witnesses who have identical accents. The application of well-replicated findings on accents could shed light on the suitability of avatars in mock-courtroom research.

Beyond standard and non-standard accents there is the issue of an accent identifying speakers as foreign-born (Derwing and Munro, 2009) making them potentially susceptible to negatively perceptions (Birney et al., 2020) depending on CO. It is not the accent that is problematic per se but rather how the listener feels about the broad geographical region from which the accent comes (Lindemann, 2011).

In the US, highest favorability ratings are given to Western European accented English speakers (Lindemann, 2005; Lippi-Green, 2011) likely because they are seen to have high status compared to those from more stigmatized countries (Dragojevic and Goatley-Soan, 2020; Lee and Fiske, 2006). US participants rate French accented English more favorably than Russian (Eichinger et al., 2009; Rakić and Steffens, 2013), Chinese or Indian (Lindemann, 2005) accented English. In a study comparing speakers of several foreign accents with GAE, Dragojevic and Goatley-Soan (2020) found that the latter were rated higher than any foreign accented speakers. Among the foreign-accented speakers, Germans received the highest status ratings, Vietnamese, Farsi, and Mandarin received lower favorability ratings and Arabic-accented English received the lowest ratings showing that listeners do not group non-standard accents homogenously. They differentiate between groups, assigning different levels of status and stigma to less desirable groups. A study assessing geographical region of origin (German, Mexican and Lebanese) and GAE and non-standard accent delivered by mock witnesses revealed that GAE speakers were viewed as more accurate, credible, prestigious and less deceptive than those who spoke with foreign accents (Frumkin, 2007).

The purpose of this study is to investigate whether, and to what extent, avatar eyewitnesses can be used in place of human eyewitnesses (mode of presentation) in jury decision-making studies relying on the known effects of accent and CO (Frumkin, 2007). Following the research cited above, we investigated whether GAE-accented witnesses would be rated most favorably, followed by witnesses with German, Mexican and Lebanese Arabic accents. GAE is extremely familiar due to much US media and entertainment in the UK. Germany is geographically close to the UK and viewed as a country with a similarly high standard of living. There is some suggestion that Arabic speakers would be less highly rated than any other (Hancock, 2007) but there is not a body of research to support this. The Mexican accent was included to shed light on possible differential ratings between UK and US participants as immigration from Mexico to the US is a concern that is not mirrored in the UK (Mateos, 2019).

Two hypotheses can be tested as regards accent and CO:

*Hypothesis 1*: The non-standard accented eyewitness will be rated less well than her GAE accented counterpart.

*Hypothesis 2*: The German eyewitnesses will be rated highest and the Mexican and Lebanese less well.

There is insufficient prior research to present a firm hypothesis regarding mode of presentation. Research question 1 concerns whether avatars and humans elicit the same favorability hierarchy based on accent and CO. This would help to establish whether avatars could replace live witnesses in mock courtroom studies. It is possible that avatars would not invoke the same reactions as humans, so that negative perceptions toward speakers with non-standard accents and against the Mexican or Lebanese speaker would be less apparent or absent for the avatars. A 2 (GAE vs. non-standard) × 2 (mode of presentation) × 3 (country of origin) Analysis of Variance examined these two hypotheses and the research question.

## 2 Methods

### 2.1 Design

This study presented participants with one of 12 videos with the same witness statement delivered by different people or avatars. There was one video for each condition of levels of accent (GAE or non-standard) × three CO (German, Mexican, Lebanese) × two modes of presentation (human or avatar). The human and avatar witness appearance matched the relevant CO by using humans from the region where the witness came from; the avatars were created to look like the human witness. Each witness was presented as a women, with similar pose, facial expression and clothing, the humans wore identical outfits and the avatars were presented with very similar clothing. The witnesses’ visual appearance was kept as similar as possible across conditions, such that the differences were only those necessary to present a visual appearance consistent with the CO. Had we presented the same witness in all conditions they could not have looked equally plausible as someone whose ethnic origin was German, Mexican, or Lebanese.

We used the matched-guise technique (Lambert et al., 1960) meaning that each witness voiced testimony in spoken English with either a GAE or German, Mexican or Lebanese accent (referred to as non-standard at the group level). The three witnesses provide identical testimony positively identifying a burglar. Each of those three witnesses were also depicted as avatars who presented the same statements with the original voices. Participants rated the perceived favorability of the witness presented to them and we examined how accent, CO and mode of presentation (i.e., avatar or human) impacted those ratings. The perceived favorability score (PFS) given to each eyewitness was calculated as the sum of the variables accuracy, credibility, confidence, prestige, trustworthiness, strength of evidence and deception (reverse scored) for each participant. The study received ethical approval from the School of Psychology human research ethics committee [university to be added after review].

### 2.2 Participants

In total, 703 psychology university students local community members, all from London, completed the study using snowball recruiting via an online link. Students sent the link to acquaintances to increase and broaden the sample. They received no compensation for participation and data collection ended in 2018. The mean age of the sample was 31.6 years (SD = 12.2) with an age range of 18–79 (68 participants did not record age). In terms of gender, 395 (61%) participants were female and 249 (39%) were male (59 participants did not record gender). Ethnicity was reported as White (including White British, White Irish and White other) = 321 (46%), Black (including African and Caribbean) = 135 (19%), Asian (including Bangladeshi, Indian, Pakistani, Chinese and other Asian) = 94 (13%), mixed = 54 (8%), any other = 24 (3%), and not known/not provided = 75 (11%). For employment status, 237 (34%) were employed full time, 237 (34%) were students, 117 (17%) were employed part-time, 43 (6%) were unemployed, and 69 (10%) were not recorded. There may have been overlap between these categories as many students, especially post-graduate, are also employed. This sample shows good representation of ethnic groups in London and spans a range of ages.

In the avatar condition there were 341 participants, mean age = 27.8 (SD = 9.3), 66% were female, and in the human condition there were 362 participants, mean age = 35.7 (SD = 13.6), 56% female. A significant difference was found between the groups in terms of age [*t*(633) = 8.58, *p* < 0.001]. Age was associated with favorability and thus would have benefitted the human condition, whereas (as will be seen) the favorability ratings were higher (non-significantly) in the avatar condition, the difference in age does not appear to have had any impact on the results.

### 2.3 Measures

**Figure 1:**
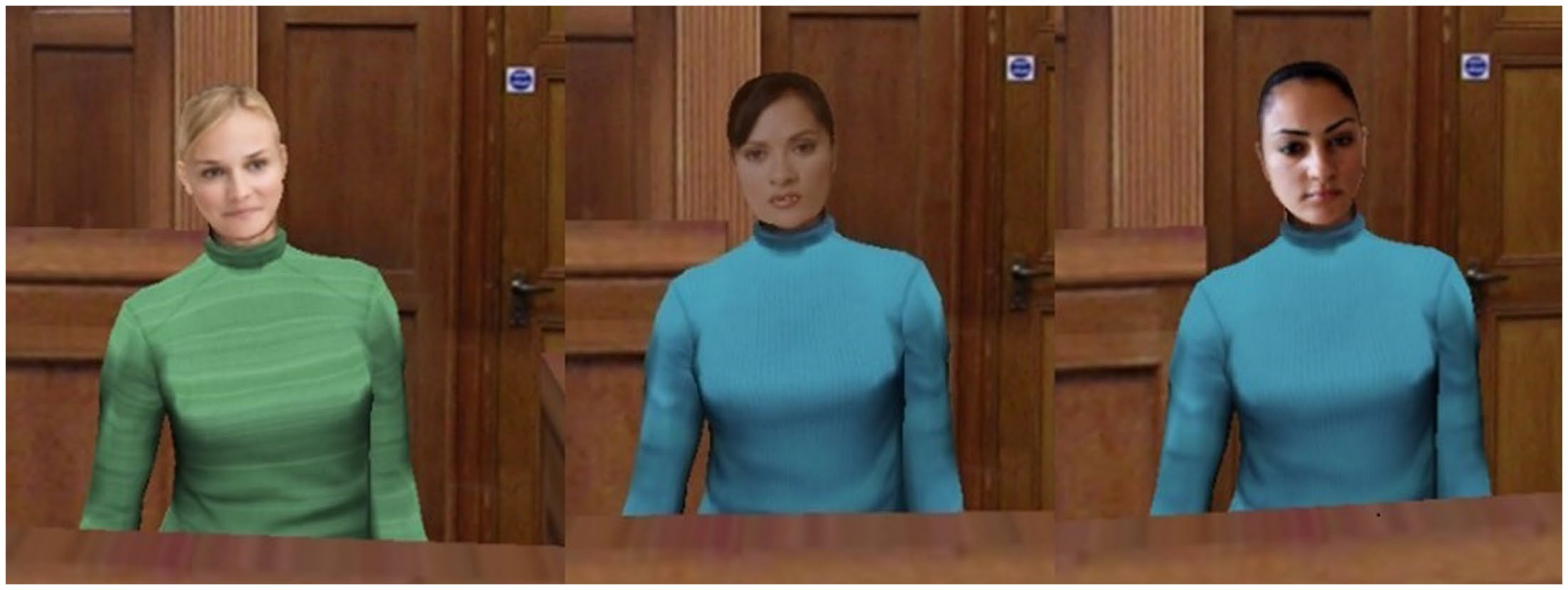
Screenshots of the three avatars and part of the courtroom (German, Mexican, and Lebanese).

Avatars (see image A) were created to appear human-like. But readily distinguishable from humans in order to avoid invoking uncanny valley effects (e.g., Mori, 1970/2012) in which a representation bearing too much human-likeness invokes sensations of eeriness or disquiet, whereas a less human-like representation does not. The avatars were designed so that their facial features, proportions and skin tone were all correct, but the avatars were clearly computer-generated (see Figure 1).

To test mode of presentation we examined the main effect of human vs. avatar witnesses. To test the effect of accent, we examined the main effect of GAE against non-standard accent. To test CO, we compared the German witnesses (combining both accents and both modes of presentation) against the Mexican (similar) and Lebanese (similar) witnesses. We also examined interactions of these factors to see whether the effect of mode of presentation would be the same across all combinations of accent and CO.

In the human condition, participants viewed the same video as used by Frumkin (2007). In each video a female is seen describing a crime she has witnessed (armed burglary). The videos were all identical apart from the witness (there were 3 different witnesses) and the accent used by the witness (there were 2 versions of each witness, one GAE condition and one non-standard accent condition) using the matched-guise technique (Lambert et al., 1960). For full details of the development of these materials please see Frumkin (2007). For most of the video the witnesses all follow the same script, apart from the start when asked to state their place of birth (the response is either Berlin, Beirut, or Mexico City dependent on ethnic background), and for the witnesses in the non-standard accent condition, they are asked to state the length of time they have lived in the US (the witness response is 5 years). We did not believe it was necessary to perform any manipulation check to see that the participant had acknowledged the CO.

In the avatar condition, participants viewed a computer animated version of the videos used by Frumkin (2007). As with the human condition, these animations were all identical, apart from the avatar used for the witness (3 different witnesses), the accent used by the witness (2 versions of each witness, GAE and non-standard) and information about their place of birth and length of time living in the US. Importantly, the avatars used the same voice recordings as used in the videos of the human witnesses. Furthermore, the avatars were designed to visually resemble the actors originally used. The avatar videos differed to the human videos slightly, as in the avatar the back of a female prosecutor is visible for a small part of the testimony (the female prosecutor is only heard in the human condition) and more of the courtroom is shown.

### 2.4 Procedure

The study was conducted online and the procedure was identical in the human and avatar conditions. After giving consent, participants were asked questions about their demographics (see Participants section). They then watched a 2-min video showing a segment of fictional eyewitness testimony taken from a criminal trial. The video watched was randomly selected from the 12 human and avatar videos. Following this, they were asked to rate the eyewitness on 11 statements. An example of a statement is “how credible do you think the witness is?” with responses given on a 10-point scale, 1 (not at all credible) and 10 (credible). Participants were asked to indicate how long the sentence should be, assuming the defendant was found guilty. The study took between 10 and 15 min to complete.

## 3 Results

Table 1 presents the raw data, including means, standard deviations, and confidence intervals for the perceived favorability score (PFS). It is notable that the range between the lowest and highest favorability ratings was 0.58 for the avatar witnesses and 0.83 for the human counterparts, indicating a wider range of reactions to the human witnesses.

**Table 1:** Mean, (SD), and 95% confidence intervals of Perceived Favorability Score (PFS) ratings by mode of presentation, country of origin, and accent.

	Avatar		Human	
	Mean (SD)	95% CI	Mean (SD)	95% CI
Lebanese – non-std	6.28 (1.61)	5.80–6.75	5.29 (1.82)	4.84–5.73
Lebanese – GAE	5.99 (1.52)	5.56–6.43	6.12 (1.99)	5.62–6.63
Mexican – non-std	6.11 (1.55)	5.69–6.54	5.45 (1.78)	5.03–5.87
Mexican – GAE	5.77 (1.64)	5.39–6.16	5.48 (1.85)	5.02–5.95
German – non-std	5.69 (1.58)	5.29–6.08	5.68 (1.69)	5.24–6.12
German – GAE	6.22 (1.48)	5.82–6.61	5.46 (1.82)	4.86–6.06
Total	5.98 (1.57)		5.58 (1.84)	

The intention was to use the dependent variable (PFS) from previous research for this analysis. The PFS was the mean of ratings of confidence, trustworthiness, accuracy, prestige, credibility, strength of evidence, and intent to deceive. However, a factor analysis suggested two factors rather than one PFS. Factor 1 includes all the PFS items except intent to deceive and the second factor includes intent to deceive. Intent to deceive was the only negatively worded item in the questionnaire and previous work has shown that positively and negatively worded items load on different factors (Zhang et al., 2016). Intent to deceive was analyzed as a separate factor.

Perceived attractiveness of the witness was positively correlated with the PFS ratings, *r*(701) = 0.32, *p* < 0.001, but not with Deception, *r*(701) = 0.05, ns, thus perceived attractiveness was entered as a covariate for the analysis on PFS as well as its interaction terms with the experimental factors.

ANCOVA was used to examine hypotheses 1 (accent) and 2 (CO), and the research question around similarity of reactions to avatars and humans. There were three between-participant experimental factors of mode of presentation (human vs. avatar), CO (German, Mexican, Lebanese), and accent (GAE vs. non-standard). Participant factors of race (White vs. non-White), and sex (female vs. male) were considered as fourth factors in the analysis. Neither participant race or sex had any main effect or interactions with any other factors, so they are not further reported.

**Figure 2:**
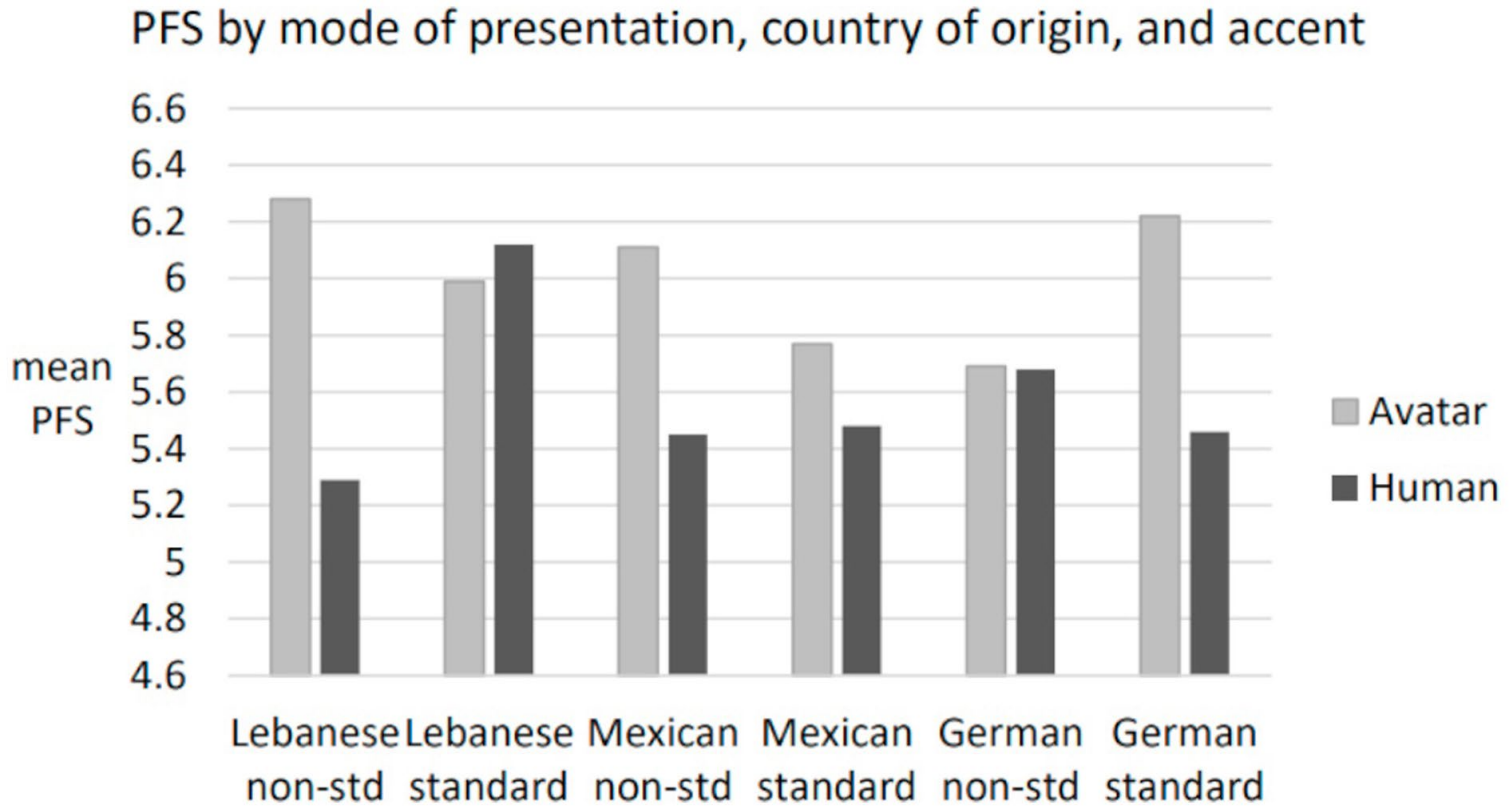
Perceived favorability score (PFS) by mode of presentation, country of origin, and accent.

ANCOVA was performed with three independent factors of mode of presentation, CO, and accent with the covariate of perceived witness attractiveness (see Table 1 and Figure 2). There was a main effect of mode of presentation [*F*(1, 690) = 10.54, *p* = 0.001, partial eta^2^ = 0.15] showing that avatars received higher PFS ratings (*M* = 5.98, SD = 1.57) compared to human witnesses (*M* = 5.58, SD = 1.84). There was no main effect of accent, *F* < 1, and no main effect of CO, *F* < 1.3.

There was a three-way interaction of mode of presentation, CO, and accent, [*F*(2, 690) = 3.90, *p* < 0.03, partial eta^2^ = 0.011]. The three-way interaction was investigated by analysis of simple effects using Bonferroni adjustment for multiple post-hoc tests. Three simple comparisons were of interest. The human Lebanese Arabic accented witness (*M* = 5.29, SD = 1.82) received lower PFS ratings compared to the same witness speaking GAE (*M* = 6.12, SD = 1.99), [*p* < 0.005] and lower ratings than her Lebanese Arabic accented avatar (*M* = 6.28, SD = 1.61), [*p* < 0.005]. In fact, the human Lebanese Arabic accented witness received the lowest ratings of all groups. In contrast, the human Lebanese witness speaking GAE (*M* = 6.12, SD = 1.99) received the third highest favorability rating of all groups. The two conclusions suggested by this pattern of results are that the human Lebanese eyewitness was rated relatively low when speaking with a Lebanese-Arabic accent and relatively high when speaking with a GAE accent. Figure 2 confirms this picture. The other contrast of note is the comparison of attractiveness between the human and avatar witnesses, which showed no significant difference, *t*(701) = 0.67, ns.

With deception as the dependent variable, the only significant effect was the main effect of mode of presentation [*F*(1,691) = 4.04, *p* < 0.05, partial eta^2^ = 0.006], showing the avatar (*M* = 3.99, SD = 2.28) was regarded as less likely to intend to deceive than the human witness (*M* = 4.30, SD = 2.42). The pattern of results was similar to the pattern observed when PFS was the dependent variable. When we ran the ANCOVA with the dependent variable used in previous research (PFS and Deception combined), the pattern of results resembled that for PFS as the dependent variable.

These results do not support hypotheses 1 or 2 as there were no main effects of accent or CO. There are two results relevant to research question 1. They are (1) that avatars were rated more favorably than human witnesses as a main effect and that (2) the Lebanese human witness with a Lebanese accent was rated less favorably than her GAE-accented counterpart, her avatar counterpart, and the Mexican and German human non-standard accented witnesses.

## 4 Discussion

Using avatars to study the influence of extralegal factors in courtroom research was an ambitious goal. Had we found no differences arising from mode of presentation, or patterns of accent and CO ratings that were similar to those found with human eyewitnesses, an argument could be made that research using avatars would be a good proxy for actual courtrooms. However, the findings from this study show that avatars and humans are perceived differently and crucially inconsistently, thus we cannot use the former in place of the latter just yet (Research Question 1).

The results showed no main effect of accent or country of origin, failing to support hypotheses 1 and 2. There was a main effect of mode of presentation and a three-way interaction showing the Lebanese human witness with a Lebanese accent was rated less favorably than all her counterparts, and less favorably than all other witnesses.

Looking first at the main effect of mode of presentation, avatars were rated significantly higher than human witnesses on PFS, and lower on intent to deceive. This may be because they are not perceived as having the imperfections of humans which could have lowered favorability ratings. It is also more natural to compare ourselves to another human as opposed to an avatar, thus we may be more willing to make stronger, harsher judgments about a person. As the avatars were rated more favorably than the humans, it might also be the case that the sample was willing to rate the newer technology more generously.

These findings are similar to those reported by Frumkin and Stone (2020) who found participants awarded harsher judgments to same-race than to other-race witnesses speaking with a non-standard accent. Similarly, participants in this study may have been more forgiving in ratings of avatars as the ‘other’ than they were with humans, who they saw as the same, i.e., human like the participant.

Second, there was a three-way interaction showing relatively low ratings for the human Lebanese witness using a Lebanese-Arabic accent but not for the other Lebanese conditions (either of the Lebanese avatars or human Lebanese GAE speaker). The Lebanese GAE-speaking witness was rated relatively highly. Participants may have assumed that the Lebanese GAE witness was more assimilated into the community as she was speaking with an accent that many of the US’s 328 million people use and that would be highly familiar to UK participants. This witness received the second highest (and very close to the highest) PFS ratings of all the conditions. The non-standard Lebanese witness may have been assumed to be non-assimilated as she was speaking with a foreign, non-native English accent (and yielded the lowest PFS ratings). Lebanese Arabic may evoke memories, or indeed contemporary thoughts, of conflicts from the Middle East or news reporting which shows those from the Middle East in an unfavorable light (Fuertes et al., 2012; Rakić and Steffens, 2013). The way someone speaks is connected to that person’s CO and influences the way the speaker is perceived (Birney et al., 2020). GAE witnesses might be assumed to have US friends and colleagues, whereas someone with a Lebanese Arabic accent may be perceived as remaining firmly within their own community. The perception of integration on the part of listeners could be particularly important for the Lebanese witness because of stereotyped beliefs about people from the Middle East. The process of assimilation (Uzun, 2023) leads to a member of the outgroup being accepted into the ingroup and therefore benefitting from ingroup favoritism. In contrast, a witness who is perceived as not being assimilated may be regarded as having rejected US values and thus had less standing as a witness. A witness with a standard GAE accent would be perceived to be well assimilated, with an accent familiar to the listener, thus eliciting higher ratings.

It is possible that the SCM provides an explanation when hearing an accent from a country that elicits a negative stereotype (Carlson and McHenry, 2006) and may be regarded as troublesome and possibly even inimical to Western interests. The SCM describes how the perceived social status of an individual influences evaluations of warmth and competence, in turn leading to negative opinions and perceptions (Fiske, 2018).

It is also worth noting that there was a greater range of favorability ratings for the human witnesses than the avatars. This might suggest that humans were perceived as less homogenous than avatars, and that finer distinctions between human witnesses were made. This seems to have been the case for the Lebanese human witness, whose favorability rating depended on accent more than for her Lebanese avatar counterpart. Alternatively, there may be a lesser tendency to give ratings based on stereotypes of people from different cultures when they are avatars. Perhaps an avatar does not invoke stereotype content to the same degree as a human. If either of these explanations are true, then courtroom research may be able to use avatars for some purposes, but not when judgments are concerned that could be affected by social, ethnic and demographic factors.

Research (e.g., de Borst and de Gelder, 2015; Donath, 2007; Felnhofer et al., 2018; Kothgassner et al., 2017; Leding et al., 2015; Nowak and Rauh, 2008; Yee et al., 2007) found that avatars who look human-like evoke similar responses to humans, though some reservations were noted. Other research, e.g., by Riedl et al. (2011), indicates attributing a mind to humans and not avatars evidencing differential information processing. The current study contributes to the literature by reporting some differences between evaluations of human and avatar witnesses.

Hypotheses 1 and 2 were not supported: there was no main effect of accent or CO. Accent has been reliably shown to affect many aspects of person perception and impression formation (Cantone et al., 2019; Gluszek and Dovidio, 2010; Lev-Ari and Keysar, 2011; Moyer, 2013) thus it is surprising that Hypothesis 1 was not supported. It was expected that there would be a main effect of accent which has been observed previously in a US sample (e.g., Frumkin, 2007). This suggests that to a UK audience, GAE was not necessarily perceived to be of higher status than non-US accents. The findings could be relevant to the debate about whether it is accent familiarity that yields higher ratings or the knowledge that the speaker is from the same group (in-group effect) as the listener.

The research points to the idea that the in-group, or solidarity component of APT, (Fuertes et al., 2012) is important as none of the witnesses were from the same group as the British listeners, explaining the lack of an overall effect of GAE vs. the non-standard accent. If higher favorability ratings were due to accent familiarity, GAE would have been higher rated as it is likely more familiar than German, Mexican, or Lebanese accents, hence these findings argue against the accent familiarity explanation. This expands the findings of other research in providing a possible rationale for ratings of accents as being those of in- and out-group speakers rather than reliant on the familiarity of the accent. The cultural association of particular non-US accents with low status, for example Mexican accented Spanish, would not apply as there are not a substantial number of immigrants from Mexico in the UK.

The hierarchy reported in Frumkin (2007) in which the German accent was rated more highly than Lebanese was not replicated in the present study, refuting Hypothesis 2. This is surprising as the German witness could be perceived as more similar to the listeners as both are from European countries compared with Mexican or Lebanese witnesses, neither of whom are European. Perhaps an explanation is that even before the 2016 Brexit vote people in the UK had mixed feelings about UK membership of the European Union (EU). Another possibility is that people make judgments on the basis of very little information (Willis and Todorov, 2006) or cultural stereotypes, thus further research on accent hierarchies is warranted.

The lack of replication has yielded some issues to consider, for example whether studies can be generalized from one country to another. Undoubtedly some of the values that people have in the US are not translatable to the UK. Perhaps the lower ratings for the Lebanese witness found in Frumkin (2007) are even stronger now, or there is something different about US and UK samples. A much larger cross-cultural study would be needed to investigate this.

### 4.1 Limitations and future directions

This study used three avatars in the role of eyewitness only. Having additional avatars with other variations (e.g., different clothing, accessories better representing native country) may have altered the results though could also have made the witnesses look like caricatures of certain groups. In this study, the only indication of CO, besides accent, was a statement about city of birth. Researchers should continue to look for factors that may influence the way eyewitnesses are perceived and whether it is accent alone, or accent alongside other features identifying the witness as being from a specific group, that effect favorability ratings.

There are limitations of a mock jury study. These include not being able to raise the stakes in decision-making sufficiently to replicate what real jury duty would be like, lack of realistic details of length of trial and using participants who have agreed to take part rather than those called for jury duty. The current study had all of these limitations and the ability to use alternate methods, for example avatars, to create a virtual courtroom, would be advantageous if further study could navigate how to do this effectively.

Future studies could use a broader sample to represent a more diverse population. The incorporation of more realistic courtroom scenarios and complex cases would improve the ecological validity of the findings.

### 4.2 Conclusion

This study was a first step in trying to determine if participants judge avatar and human eyewitnesses similarly. A consistent perceived favorability rating pattern across the accent and CO conditions with the different modes of presentation would have provided promising findings to expand extralegal research possibilities. Instead, this study has shown that mock jurors do not rate witnesses as equal even when everything except whether the witness is human or avatar is held constant so particular patterns of prejudice that apply to human witnesses may not apply to avatars. This is a warning against the use of avatars in this sort of research at present. Mock courtroom research is time consuming so avenues investigating how virtual reality may benefit understandings of jury decision-making is useful.

## Data Availability

The raw data supporting the conclusions of this article will be made available by the authors, without undue reservation.
